# Mantle Cloaks Based on the Frequency Selective Metasurfaces Designed by Bayesian Optimization

**DOI:** 10.1038/s41598-018-32167-x

**Published:** 2018-09-19

**Authors:** F. F. Qin, Z. Z. Liu, Q. Zhang, H. Zhang, J. J. Xiao

**Affiliations:** 1 Shenzhen Engineering Laboratory of Aerospace Detection and Imaging, College of Electronic and Information Engineering, Shenzhen Graduate School, Harbin Institute of Technology, Shenzhen, 518055 Guangdong, China; 2 SZU-NUS Collaborative Innovation Center for Optoelectronic Science and Technology, Key Laboratory of Optoelectronic Devices and Systems of Ministry of Education and Guangdong Province, College of Optoelectronic Engineering, Shenzhen University, Shenzhen, 518060 P. R. China

## Abstract

We propose a full optimization procedure for designing mantle cloaks enclosing arbitrary objects, using sub-wavelength conformal frequency selective surface (FSS). Rely on the scattering cancellation principle of mantle cloak characterized by an average surface reactance, a personal computer can achieve this design procedure. By combing a Bayesian optimization (BO) with an electromagnetic solver, we can automatically find the optimal parameters of a conformal mantle cloak which can nearly cancel the scattering from the enclosed objects. It is shown that the results obtained by our method coincide with those from a rigorous analytical model and the numerical results by full parametric scanning. The proposed methodology opens up a new route for realizing ultra-wideband illusion scattering of electromagnetic wave, which is important for stealth and microwave applications.

## Introduction

In the past decade, much attention has been paid to the concept and realization of electromagnetic cloaks, which is one of the most interesting applications of metamaterials and metasurfaces^1–9^. Coordinate transformation cloaking method^10–12^ is arguably the most successful approach that has been experimentally realized in radio frequency^11^, near infrared, and the visible^7,13–15^. The principle of this technique is that by utilizing specific anisotropy and inhomogeneity profiles of the surrounding metamaterials (cloak) around an object, the electromagnetic wave inside the cloak can be bent and make the object effectively invisible. Soon later, several alternative approaches such as plasmonic cloaking^16^ and cylindrical transmission-line cloaking were proposed. However, all the above mentioned cloaking techniques rely on bulk metamaterials which are not only difficult to fabricate but also have a relatively large size compared with that of the region to be cloaked. These drawbacks greatly hinder the practical applications of these cloaking techniques.

Recently, a different cloaking method named scattering cancellation technique^17–22^ has been proposed to overcome these issues. In this framework, the scattered fields from a given object are cancelled through generating “anti-phase” currents on a thin surface around the object. Once the required average surface reactance of the object has been confirmed, the specifically designed metasurface of the mantle cloak can be made by adopting the proper shape and geometry of the unit cell.

For some simple objects with canonical shapes, such as sphere and cylinder, one can use a rigorous analytical model to design the mantle cloaks^16,19–25^. But for more complicated geometries, theoretical or semi-theoretical analysis becomes difficult or even impossible. In such cases, we have to design the corresponding mantle cloaks with the aid of numerical calculations. When the designed metasurfaces only have one or two parameters to be determined, the simulations can be performed with brute-force parametric scanning. However, in many situations we need to consider much more correlative parameters that are usually high-dimensional and complex (mostly nonlinear). In addition, other issues such as fabrication requirement of the devices, attrition rate of the material should also be taken into account. Considering all these factors, direct parametric scanning in simulations is inadvisable and some sophisticated but efficient optimization algorithms are highly demanding^26,27^.

The ultimate aim of optimization algorithms is to provide efficient numerical algorithms that can quickly find the minimal or maximum of a deterministic black-box function $f$, which may satisfy one or more of the following criteria: (1) it does not have a closed-form expression, being expensive to evaluate and (2) does not have easily available derivatives (non-convex). There are many methods for optimizing over parameters settings, ranging from simplistic procedures like grid or random search^28–30^, to more sophisticated model-based approaches using random forests^31^ or Gaussian process (GP)^32^. Among these optimization algorithms, Bayesian optimization (BO) is a well recognized algorithm for solving global optimization problems with non-convex black-box function^33–38^. There are two aspects of advantages that make BO different from other optimization procedures. It involves a probabilistic model for the unknown objective function to be optimized and exploits such probabilistic model to acquire extra points to further modify the objective function. GP is the most commonly used probabilistic model in BO, due to its simplicity and flexibility in terms of conditioning and inference.

In this work, we combine the BO algorithm with full-wave electromagnetic simulation to efficiently design mantle cloaks using metasurfaces composed of sub-wavelength frequency selective surface (FSS) elements. Specifically, we build up a bridge between the BO algorithm and commercial electromagnetic packages, e.g, COMSOL Multiphysics^39^, to automatically find the optimal parameters for FSS elements that can substantially reduce the overall scattering from a target object. As a benchmark, we firstly design a mantle cloak for a 2D cylinder. It is shown that the results achieved by our method coincide with the analytical results from a rigorous analytical model based on the Lorenz-Mie scattering theory^40,41^. We also confirm that the results obtained by our method and from parametric scanning are consistent. We then further demonstrate that the proposed method can successfully design mantle cloaks based on metasurface for other two- (2D) and three-dimensional (3D) geometries that are impossible to be theoretically analyzed.

## Results and Discussion

Supposed the size of the FSS elements is far less than the operating wavelength, the FSS can be effectively described with an equivalent surface impendence ${Z}_{s}={R}_{s}-i{X}_{s}$, where ${R}_{s}$ is the surface resistance associated with losses and ${X}_{s}$ relates to the stored energy, which can be either inductive or capacitive, depending on the FSS structure. For simplicity, here we only consider the lossless case with ${R}_{s}=0$. The limiting case ${X}_{s}\to \pm \,\infty$ corresponds to a bare object without cloak, as the metasurface has no interaction with the scattering field of the object. Based on the electromagnetic multipole theory, the total scattering cross section (SCS) of a scatter can be expressed as^42^

1 $${C}_{s}=\frac{\pi }{{k}^{2}}\sum _{l=1}^{\infty }\sum _{m=-l}^{l}(2l+1)[{|{\alpha }_{E}(l,m)|}^{2}+{|{\alpha }_{M}(l,m)|}^{2}]$$

where *k* is the wave number; ${\alpha }_{E}(l,m)$ and ${\alpha }_{M}(l,m)$ are the multipole scattering coefficients. For an infinite long cylinder or a sphere with mantle cloaks, the scattering coefficients can be analytically calculated by forcing a discontinuity of the tangential magnetic field on the surface of the cloak, and they are functions of the frequency, ${X}_{s}$ of the cloak, the thickness and dielectric constant of the material filled between the cloak and the object^19–22^. This holds true for more complicated objects though the explicit functions cannot be obtained analytically.

By proper selection of the FSS structure, the thickness and dielectric constant of the material filled between the cloak and the object, the multipole scattering coefficients may be made zero, which indicates drastic suppression of the scattering for a given object for any angle of incidence and observation, making it almost completely undetectable, i.e., invisible, at the frequency of interest. The physical mechanism behind the scattering elimination of mantle cloak is that the destructive interference between the fields induced by the FSS elements and that scattered by the object happens at all angles.

**Figure 1:**
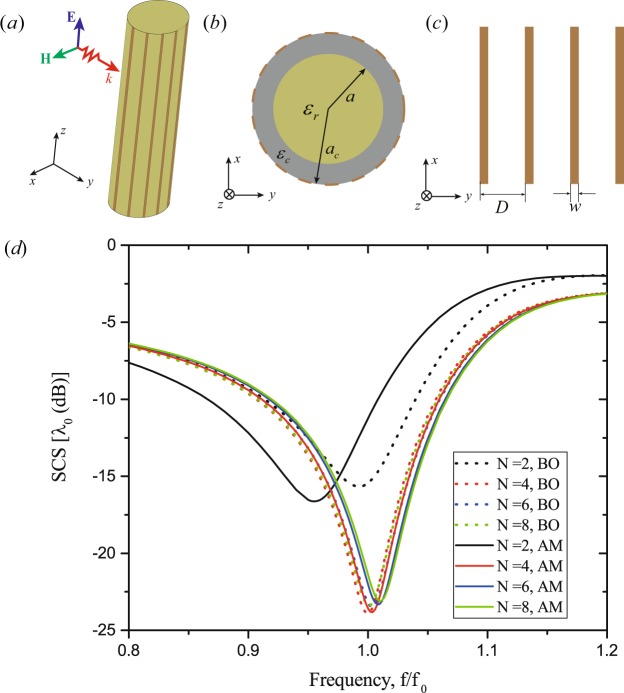
The mantle cloaks design for the infinite dielectric cylinder. (**a**) 3D view of the cylinder with a conformal array of vertical strips, (**b**) top-view of (**a,c**) periodic grid of the planar vertical strips, (**d**) the SCSs curves of the inductive strips for different number of strips obtained by the Bayesian optimization (BO) and actual analytical model (AM).

To demonstrate the automatic design of mantle cloaks based on the BO procedure (see the BO in the Method and the detailed optimization process in the Supplementary Information), we begin with an example for a dielectric infinite cylinder with relative permittivity ${\varepsilon }_{r}=8$ and diameter $2a={\lambda }_{0}/6$ (${\lambda }_{0}$ is the free-space wavelength at the design frequency ${f}_{0}$). Mantle cloaks for dielectric cylinder have been realized using different structures and a FSS with inductive surface reactance is usually employed^18,23^. As shown in Fig. 1(a), here we use an ultrathin concentric cloaking metasurface composed of 1D periodic array of metallic vertical strips, which is commonly used to provide an inductive surface reactance^43,44^. The concentric cloaking metasurface is directly glued on the cylinder, *e.g*. with radius ${a}_{c}=a$ [Fig. 1(b)]. From the analytical results in ref.^21^ [Appendix Eq. (A2)], it is known that the grid impedance of the metasurface is frequency dispersive and determined by the grid parameters, including the period $D$ ($D=2\pi {a}_{c}/N$, where $N$ is the total number of the strips) and width $w$ [Fig. 1(c)].

**Figure 2:**
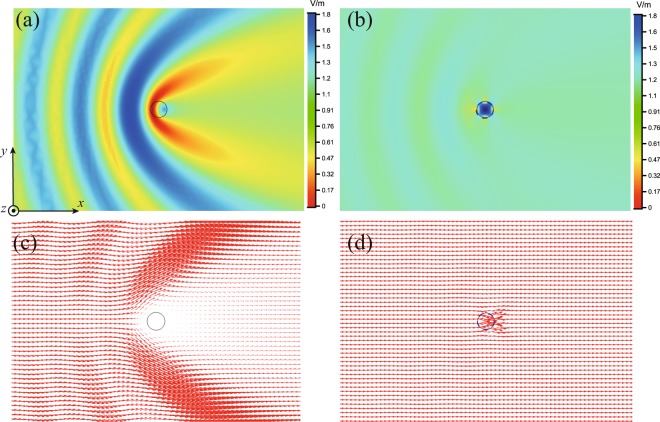
Full-wave numerical results for the BO final solution of the circular cylindrical structure. Magnitude of the electric fields on the $xy$-plane: (**a**) without the cloak and (**b**) with the vertical strip cloak. Vector power-flow distributions on the $xy$-plane: (**c**) without the cloak and (**d**) with the vertical strip cloak.

The optimal parameters for the mantle cloaks obtained by the analytical model and the BO algorithm are summarized in Table 1 and the corresponding SCSs are shown in Fig. 1(d). It can be seen that the SCSs obtained by the BO algorithm are at the same level to that by the analytical model, and as $N$ increases, the optimal parameters obtained by the numerical results are much closer to that of the analytical results (see Table S1 in Supplementary Information). To further show the effect of the mantle cloak designed by the BO algorithm, in Fig. 2 we compare the electric field amplitude and power-flow distributions of the cylinder cloaked and uncloaked ($N=4$), respectively. The incident wave travels along the $+x$ direction and its polarization direction is parallel to the $z$ axis. In absence of the mantle cloak, Fig. 2(a) shows that the electric field around the cylinder is apparently disturbed due to the significant scattering. On the contrary, when the mantle cloak is present a uniform electric field distribution is obtained as if the cylinder is not present, as seen in Fig. 2(b). Figure 2(c,d) show the power-flow distribution for the uncloaked and cloaked dielectric cylinder, respectively. For the no cloak case in Fig. 2(c), it’s obvious that the power-flow distribution is deranged by the cylinder and a shadow region appears in the forward direction. It is seen in Fig. 2(d) that the energy drifted around and through the cloak is not subjected to much perturbation, which indicates that the object looks not existing.

**Table 1:** The optimal parameters obtained by the analytical model and BO algorithm for the case of 1D periodic array of metallic vertical strips around the cylinder.

Method	*N*	*ε*_*r*_	Radius (*a*_*c*_ = *a*)	Periodicity *D*	Width *w*
Analytical Model (AM)	2	8	*λ*_0_/12	*λ*_0_/4	*λ*_0_/25
	4	8	*λ*_0_/12	*λ*_0_/8	*λ*_0_/198
	6	8	*λ*_0_/12	*λ*_0_/12	*λ*_0_/1200
	8	8	*λ*_0_/12	*λ*_0_/16	*λ*_0_/6478
BO Algorithm (BO)	2	8	*λ*_0_/12	*λ*_0_/4	*λ*_0_/21
	4	8	*λ*_0_/12	*λ*_0_/8	*λ*_0_/210
	6	8	*λ*_0_/12	*λ*_0_/12	*λ*_0_/1280
	8	8	*λ*_0_/12	*λ*_0_/16	*λ*_0_/7580

With the above discussions on Figs 1 and 2, we have basically shown the effectiveness of our mantle cloak design method based on the BO algorithm. It should be noted that although using the accurate analytical model, one can quickly find the satisfied FSS element parameters for some canonical objects like the infinite cylinder in Fig. 1, but for objects of random structure, it’s hard or impossible to use the same method. In the following we will demonstrate that, combining the fields in and around the objects, we can use the BO algorithm to precisely find the required FSS element parameters of mantle cloaks that can drastically suppress the scattering from more complicated objects.

**Figure 3:**
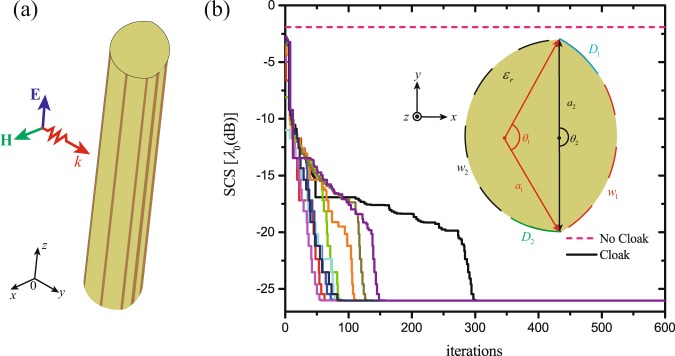
The mantle cloak for a column of random shape with a TM-polarized plane wave at normal incidence. (**a**) 3D view of the infinite dielectric column of random shape with a conformal array of vertical strips, (**b**) The 10 optimization runs with different initial choices of candidates, where the insets show the schematic top view of (**a**). The 10 runs of the BO method find the same solution: ${w}_{1}={\lambda }_{0}/2922$ and ${w}_{2}={\lambda }_{0}/655$.

Without loss of generality, we consider a dielectric object composed of two cylinders with different radius ${a}_{1}=\sqrt{3}{\lambda }_{0}/20$ and ${a}_{2}={\lambda }_{0}/10$. The structure is shown in Fig. 3(a) and the cross section is given in the inset of Fig. 3(b). The relative permittivity of the object is ${\varepsilon }_{r}=10$ and an ultrathin mantle cloak clings to the object surface. For simply and according to the curvature of the object, we choose two different periods of vertical strips with ${D}_{1}=2\pi {a}_{1}/9$ and ${D}_{2}=\pi {a}_{2}/3$, respectively. This then allows two free parameters, ${w}_{1}$ and ${w}_{2}$ for the optimization. To test the performance of the BO algorithm, 10 rounds of optimization are conducted with different initial choices of 6 candidates. As Fig. 3(b) shows, all optimization procedures have the SCS decreasing rapidly during the inception phase and then tend to converge to the same level with the optimal parameters of ${w}_{1}={\lambda }_{0}/2922$ and ${w}_{2}={\lambda }_{0}/655$, showing the high efficiency of the BO algorithm. Figure 3(b) also shows that there is a huge drop in the scattering from the object with cloaked, compared to the case of uncloaked (horizontal dashed line).

**Figure 4:**
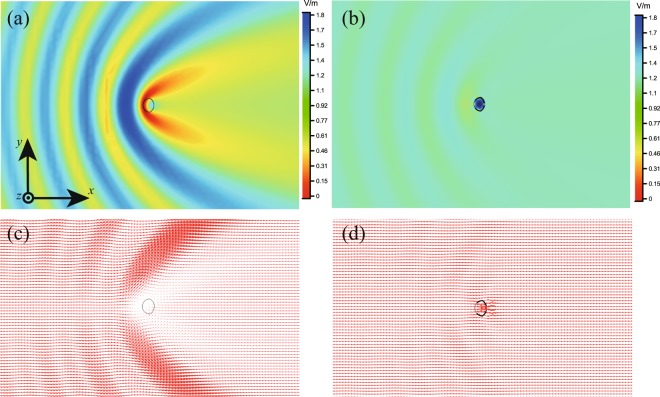
Full-wave numerical results for the BO final solution of the non-circular cylindrical structure. (**a–d**) are similar to those in Fig. 2.

Figure 4 shows the corresponding electric field amplitude and the power-flow distribution on the (*x-y*) plane for the cases of dielectric object without and with the mantle cloak, respectively. Similar to results in Fig. 2, it is seen that the mantle cloak can significantly reduce the field and power-flow disturbance around the object caused by the strong scattering.

**Figure 5:**
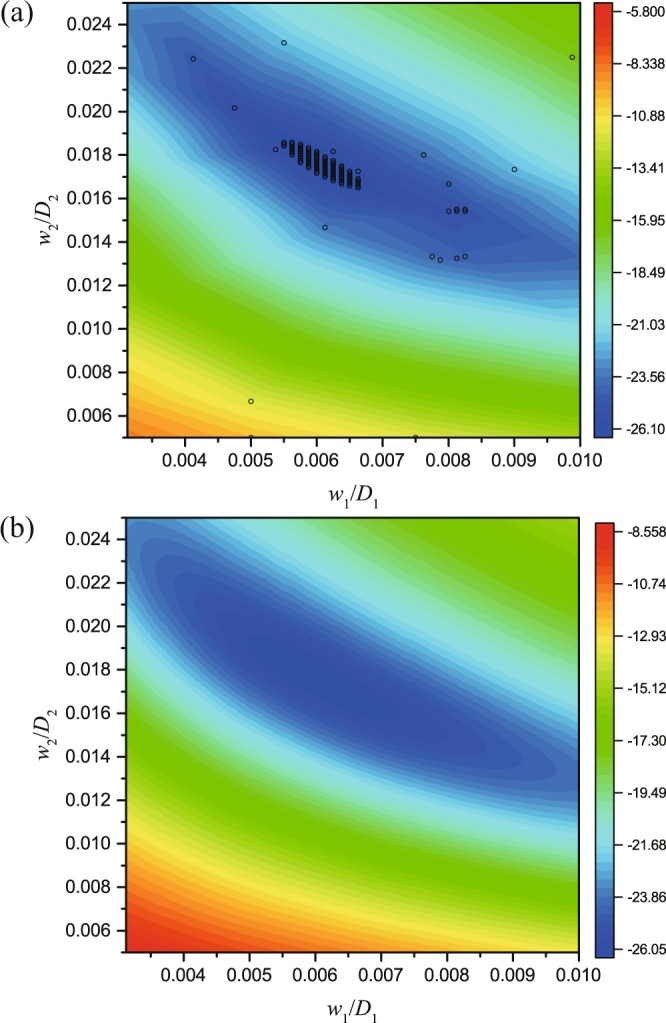
Scattering cross section distributions obtained from (**a**) the BO method and (**b**) parameter scan.

Figure 5(a) shows the local distribution of the Gaussian regression around the optimal parameters. The candidates acquired during the optimization process are depicted by circles. In this low dimensional space, it is viable to use the parametric scanning method to find the proper values in the full parameter space with acceptable computational costs. Figure 5(b) shows the brute force results which agree well with the prediction from the BO algorithm [Fig. 5(a)]. Besides, we also show the global distributions of SCSs obtained from the BO algorithm and pure numerical parametric scanning in the full parameter space (see Fig. S1 in Supplementary Information).

**Figure 6:**
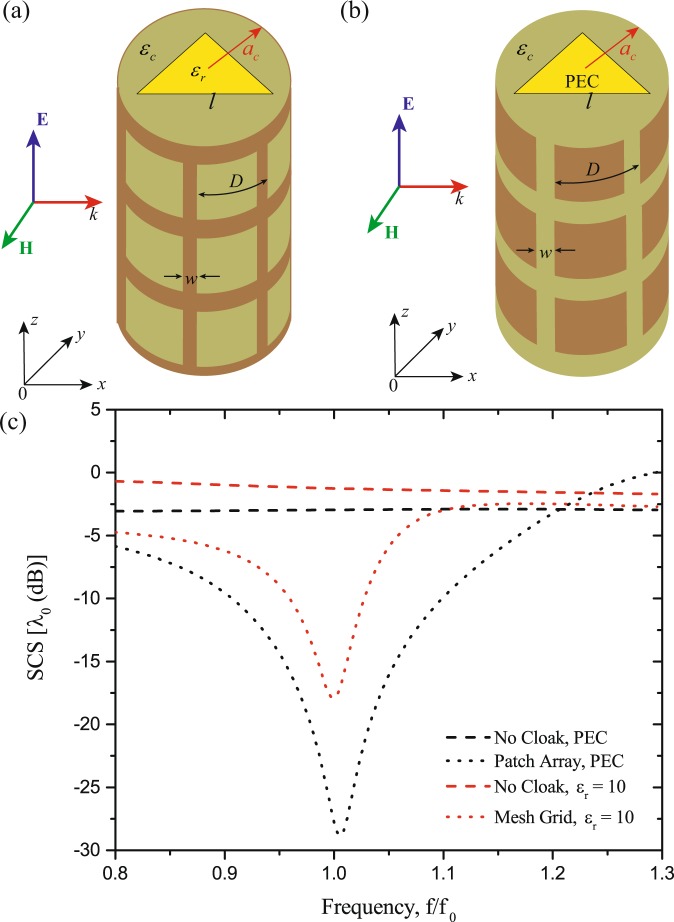
3D view of the objects. (**a**) Dielectric object and (**b**) conductor object with a conformal array of 2D sub-wavelength periodic elements, (**c**) SCS for the optimized prisms with and without cloak.

**Figure 7:**
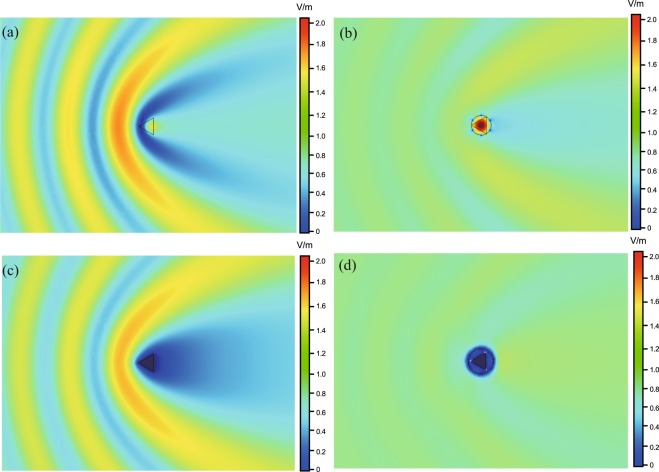
Full-wave numerical results of magnitude of the electric fields on the $xy$-plane. (**a**) Without the cloak and (**b**) with the mesh grid cloak for a dielectric prism: (**c**) without the cloak and (**d**) with the patch array cloak for a conductor prism.

Analogous to the previous example, we next show an example of designing a mantle cloak with 2D sub-wavelength periodic elements. Let us consider the case of a 2D equilateral triangle prism with the side length $l=\sqrt{3}{\lambda }_{0}/10$, illuminated by a normal incident TM plane wave. The radius of the cylinder mantle cloak is denoted by ${a}_{c}$ and the medium between the dielectric prism and the mantle cloak is a dielectric with relative permittivity ${\varepsilon }_{c}$. Here two different types of prism are concerned: a dielectric prism with relative permittivity ${\varepsilon }_{r}=10$ [see Fig. 6(a)] and a perfectly electric conducting (PEC) prism [see Fig. 6(b)]. The optimal parameters obtained by the BO algorithm and the corresponding SCS spectra are given in Table 2 and Fig. 6(c), respectively. Clearly, strong scattering cancellations are achieved with the optimal parameters when compared to the original system without the mantle cloak. Figure 7(a,b) show the amplitude distribution of the total electric field for the cases of the uncloaked and cloaked dielectric prism, respectively. It is seen that the mantle cloak makes the prism ‘disappeared’ for the incident electromagnetic wave. Figure 7(c,d) show the similar results as in Fig. 7(a,b) but for the case of PEC prism. Besides, the corresponding far-field radiation patterns for the cases of the uncloaked and cloaked prisms validate that the suppression of the scattered field happens at all observation angles (see Fig. S2 in Supplementary Information).

**Table 2:** The optimal parameters of the mantle cloaks calculated by the BO algorithm for the cases of dielectric prim and conducting prism.

*ε*_*r*_	Length *l*	Radius *a*_*c*_	*N*	Periodicity *D*	Width *w*	*ε*_*c*_
10	$\sqrt{3}$*λ*_0_/10	*λ*_0_/10	6	*λ*_0_/10	*λ*_0_/953	1.49
PEC	$\sqrt{3}$*λ*_0_/10	*λ*_0_/8	6	*λ*_0_/8	*λ*_0_/166	2

**Figure 8:**
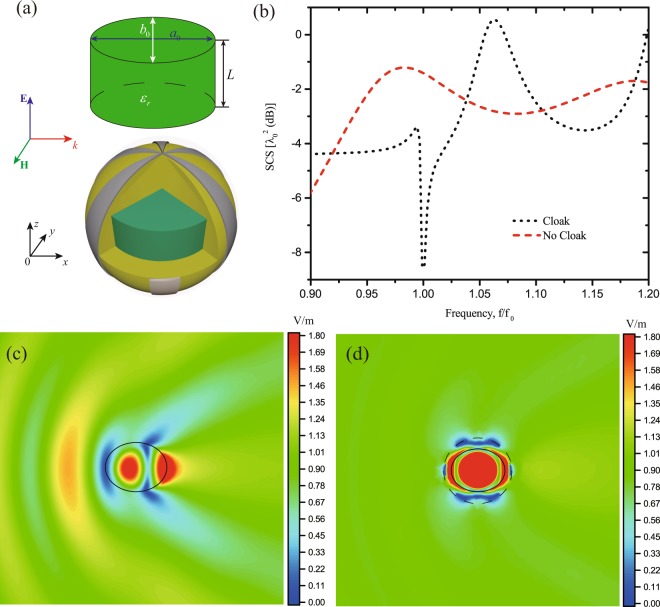
(**a**) 3D view of the elliptical cylinder and it’s covered by a sphere mantle cloak with radius ${a}_{c}$, (**b**) SCSs for the optimized elliptical cylinder with and without cloak. Full-wave numerical results of magnitude distribution of the **E**-field on the *xy*-plane for the elliptical cylinder: (**c**) without the cloak and (**d**) with cloak.

Finally, we apply the BO algorithm to a 3D mantle cloaking design, i.e., a finite length elliptical cylinder under plane wave illumination. Figure 8(a) shows the dielectric elliptical cylinder with finite length $L={\lambda }_{0}/5$, relative permittivity ${\varepsilon }_{r}=10$, geometrical dimensions ${a}_{0}={\lambda }_{0}/5$ and ${b}_{0}={\lambda }_{0}/6$, covered by a spherical mantle cloak with radius ${a}_{c}$. The plane wave travels along the $+x$-direction with electric field polarized in $z$-axis. Similar to the 2D cases, we have used the BO algorithm to design the cloak and the optimal parameters are shown in Table 3. Figure 8(b) plots the SCSs of the elliptical cylinder with and without the mantle cloak. Strong total scattering reduction appears around the frequency of interest. Figure 8(c,d) show the magnitude distribution of the ${\bf{E}}$-field on the $xy$ plane for the object without and with cloak, respectively. It is noticed that the mantle cloak indeed restrains the scattering from the object, whereas in absence of the cloak, the field distribution around the object is deranged and scattered in all directions.

**Table 3:** The optimal parameters of the mantle cloak obtained by the BO algorithm for a finite length elliptical cylinder.

Long axis *a*_0_	Short axis *b*_0_	*ε*_*r*_	Height *L*	Radius *a*_*c*_	*N*	*ε*_*c*_	Petal radians *θ* (rad)
*λ*_0_/5	*λ*_0_/6	10	*λ*_0_/5	*23λ*_0_/100	6	1	0.34

## Conclusions

Combing the BO algorithm with a full-wave electromagnetic solver, we present an automatic design procedure for mantle cloaks of both 2D and 3D objects. The feasibility of the proposed optimization approach is firstly verified by comparing the results to those calculated by the analytical model for an infinite cylinder. We further show several examples to design mantle cloaks for more complicated 2D and 3D objects. These results confirm that the mantle cloaks designed by the proposed approach can substantially reduce the scattering of the target object at the interested frequency, indicating the versatility of the approach. Our scheme has the advantage of great flexibility and strong applicability, and may be extended to a variety of applications, such as cloaked sensing and non-invasive probing^45,46^.

## Method

### The Bayesian optimization procedure

BO algorithm addresses the general problem of identifying a global maximizer (or minimizer) of an unknown objective function $f(x)$:

2 $${x}^{\ast }=\,\mathop{\text{arg}\,\min }\limits_{x\in {{\rm X}}}f(x)$$

where ${{\rm X}}$ is the $d$-dimensional design space of interest (${{\rm X}}\subset {{\mathbb{R}}}^{d}$). The design space may be a *d*-dimensional hypercube or a general constraint parameter space. In general, BO algorithm has two key ingredients. The first ingredient is a probabilistic surrogate model, which consists of a prior distribution that captures our beliefs about the behavior of the unknown objective function and an observation model that describes the data generation mechanism. Due to its simplicity and flexibility, GP has proven to be useful surrogate models for computer experiments. The second one is an acquisition function which is calculated from the posterior distribution of the unknown objective function. It is designed to measure the potential of unobserved inputs for finding the optimum with relatively a few iterations.

A GP, as a flexible Bayesian nonparametric model, provides a full probabilistic model of the data, and allows one to compute not only the model prediction at input points but also to quantify the uncertainty in the predictions. It can be defined by a mean function $\mu :{{\rm X}}\to {\mathbb{R}}$ and a kernel function $k:{{\rm X}}\times {{\rm X}}\to {\mathbb{R}}$ which is positive definite and describes the covariance of the process. The probability distribution of the tuple ${\bf{Y}}=(f({x}_{1}),\cdots ,f({x}_{N}))$ for any *N* points ${x}_{1},\cdots ,{x}_{N}\in {{\rm X}}$ is given by

3 $$P({\bf{Y}})=\frac{1}{{(2\pi )}^{N/2}{|{\boldsymbol{\Sigma }}|}^{1/2}}\exp [-\,\frac{1}{2}{({\bf{Y}}-{\boldsymbol{\mu }})}^{T}{{\boldsymbol{\Sigma }}}^{-1}({\bf{Y}}-{\boldsymbol{\mu }})]$$

where the mean values are defined as ${\boldsymbol{\mu }}={[\mu ({x}_{1}),\cdots ,\mu ({x}_{N})]}^{T}$ and the covariance matrix is ${\boldsymbol{\Sigma }}={[k({x}_{i},{x}_{j})]}_{i,j}$. By choosing different kernels $k({\rm{x}},{\rm{x}}^{\prime} )$, a large class of random functions can be described. In this paper, we consider the square exponential kernel

4 $$k({\rm{x}},{\rm{x}}^{\prime} )={\sigma }^{2}\exp (\frac{1}{2}\sum _{i=1}^{d}\frac{{({x}_{i}-{x}_{i}^{^{\prime} })}^{2}}{{l}_{d}^{2}})$$

which is widely used in practice. The hyper-parameters of the Gaussian process $\omega =(\sigma ,{l}_{1},\cdots ,{l}_{d})$, including standard deviation $\sigma$ and describing the length scales of the parameters ${l}_{1},{l}_{2},\cdots$ define higher level concepts about the model such as complexity, capacity to learn, rate of convergence, etc. And the optimal hyper-parameters lead to better efficiency, fast convergence and better results overall^44^.

For the choice of the next point to be evaluated, we use an acquisition function that enables active learning of the unknown objective function. Commonly used acquisition functions include the upper confidence bound (UCB) and expected improvement (EI). For efficiency, in this work we consider BO algorithm with EI

5 $$\alpha (x,{y}_{{\rm{\min }}})={\mathbb{E}}[\,{\rm{\max }}(0,{y}_{{\rm{\min }}}-f(x))]$$

where $f(x)$ is the statistical prediction (Gaussian distribution) of the unknown objective function at the position $x$.

## Electronic supplementary material

supplementary information
