# Supplementary material for: Mantle Cloaks Based on the Frequency Selective Metasurfaces Designed by Bayesian Optimization
Source: Sci Rep. 2018 Sep 19;8:14033. doi: 10.1038/s41598-018-32167-x (PMC6145948; doi:10.1038/s41598-018-32167-x)
Supplement: Supplementary file 1 — supplementary information [file 41598_2018_32167_MOESM1_ESM.docx]

Supporting Information for:

**Mantle Cloaks Based on the Frequency Selective Metasurfaces Designed by Bayesian Optimization**

**F. F. Qin^1^, Z. Z. Liu^1^, Q. Zhang^1^, H. Zhang^2^, and J. J. Xiao^1^,***

^1^Shenzhen Engineering Laboratory of Aerospace Detection and Imaging, College of Electronic and Information Engineering, Shenzhen Graduate School, Harbin Institute of Technology, Shenzhen 518055, Guangdong, China

*2SZU-NUS Collaborative Innovation Center for Optoelectronic*

*Science and Technology*

*Key Laboratory of Optoelectronic Devices and Systems of*

*Ministry of Education and Guangdong Province*

*College of Optoelectronic Engineering*

*Shenzhen University*

*Shenzhen 518060, P. R. China*

*^2^SZU-NUS Collaborative Innovation Center for Optoelectronic Science and Technology, Key Laboratory of Optoelectronic Devices and Systems of Ministry of Education and Guangdong Province, College of Optoelectronic Engineering, Shenzhen University, Shenzhen 518060, P. R. China*

^*^Corresponding author: [eiexiao@hitsz.edu.cn](mailto:eiexiao@hitsz.edu.cn)

**Table S1. The difference $\left|\Delta w\right|$between the optimal parameter$w$obtained by the analytical model and BO** **algorithm for the case of 1D periodic array of metallic vertical strips around the cylinder**

| *N* | Width (BO)  *w* | Width (AM)  *w* | $\left\vert\Delta w\right\vert$ |
| --- | --- | --- | --- |
| 2 | *λ*_0_/21 | *λ*_0_/25 | *λ*_0_/132 |
| 4 | *λ*_0_/210 | *λ*_0_/198 | *λ*_0_/3465 |
| 6 | *λ*_0_/1280 | *λ*_0_/1200 | *λ*_0_/19200 |
| 8 | *λ*_0_/7580 | *λ*_0_/6478 | *λ*_0_/44558 |

**The optimization process**

A connection is established between BO algorithm and CMOSL to quickly achieve the optimal parameters for the mantle cloak design. Figure S1 describes the detailed optimization process of the connection between BO algorithm and COMSOL. Here, Matlab is used as a connection between Python and COMSOL. BO algorithm run in Python can generate initial population and obtain the next candidate according to the acquisition function. Then Python calls Matlab, and Matlab calls COMSOL to update the parameters of the mantle cloak. COMSOL simulates the new candidate and output the optimization function$\sigma_{SCS}$. If the value of the$\sigma_{SCS}$cannot meet the termination condition (such as$\sigma_{SCS}$ falls by 80%), the program will update the optimal parameters for next iteration


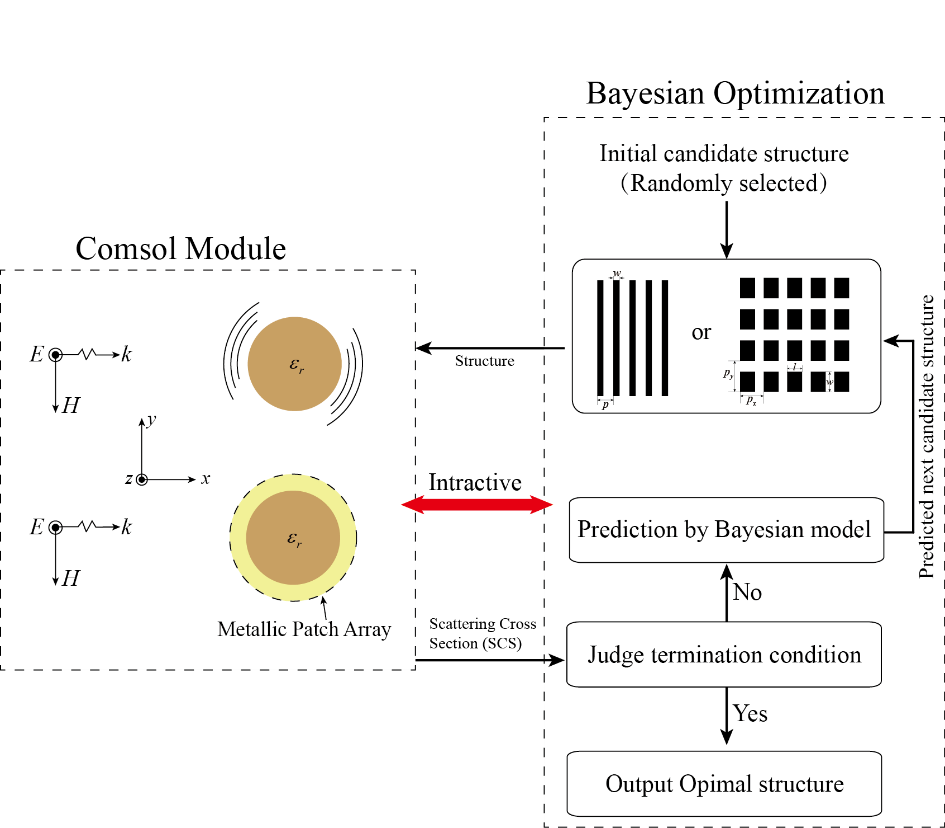


Figure S1 Flowchart of the connection between BO algorithm and COMSOL.


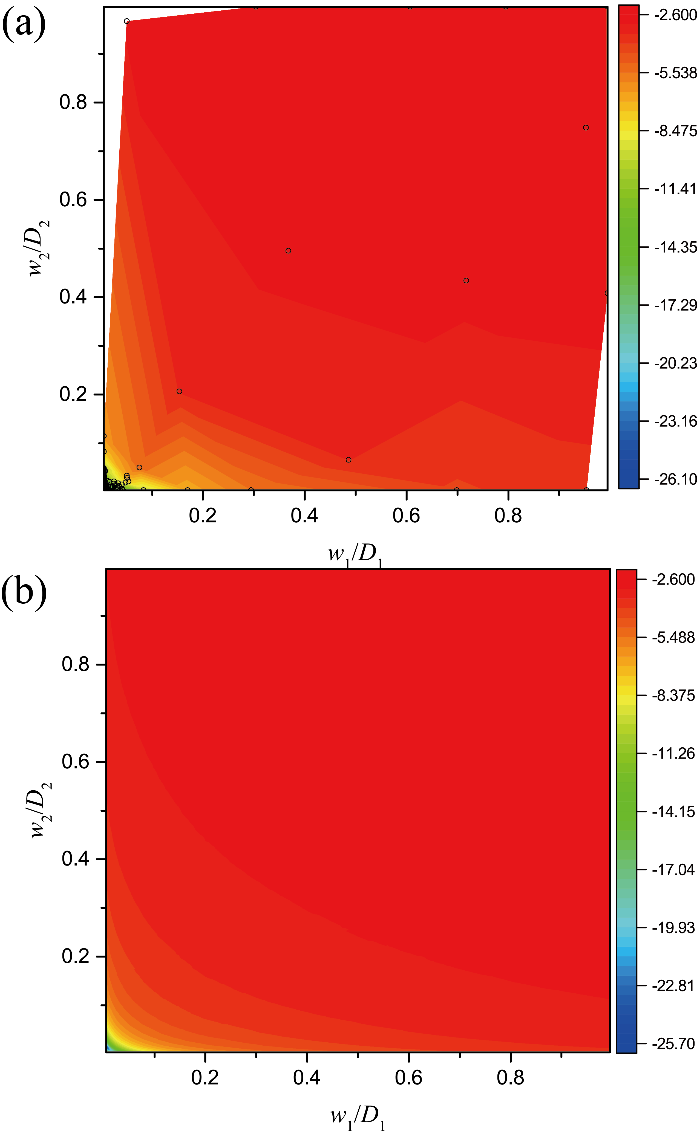


Figure S2 The global distributions of SCSs calculated by the BO algorithm (a) and pure parameter scanning (b) for a dielectric infinite object composed of two cylinders.


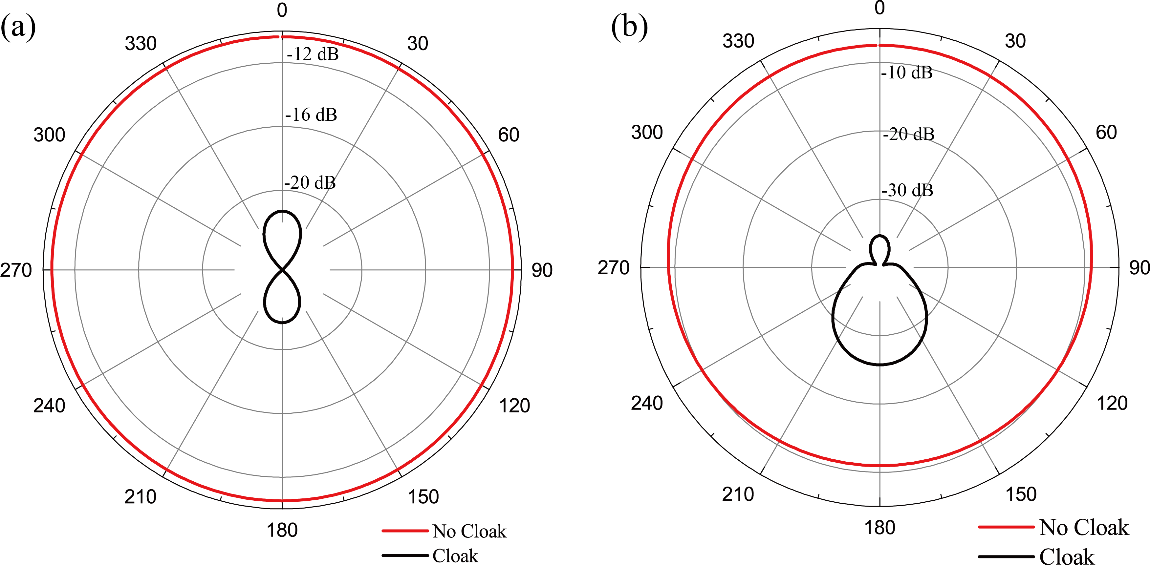


Figures S3 Far-field radiation patterns for the dielectric prism (e) and conducted prism (f) with and without the cloak.
